# Complete genome sequence of a methicillin-resistant *Staphylococcus lugdunensis* strain and characteristics of its staphylococcal cassette chromosome *mec*

**DOI:** 10.1038/s41598-020-65632-7

**Published:** 2020-05-26

**Authors:** Rie Shibuya, Yuki Uehara, Tadashi Baba, Kuniko Teruya, Kazuhito Satou, Takashi Hirano, Teruo Kirikae, Keiichi Hiramatsu

**Affiliations:** 1 Department of Microbiology, Juntendo University Graduate School of Medicine, Tokyo, Japan; 2 Department of Clinical Laboratory, Saiseikai Yokohamashi Tobu Hospital, Yokohama, Japan; 3 Center of Excellence for Infection Control Science, Graduate School of Medicine, Juntendo University, Tokyo, Japan; 4 Department of Clinical Laboratory, St. Luke’s International hospital, Tokyo, Japan; 5 AVSS Co., Ltd., Nagasaki, Japan; 6 Department of Genome Medicine, National Center for Child Health and Development, Tokyo, Japan; 7 SENTAN Pharma, Inc., Fukuoka, Japan

**Keywords:** Microbiology, Molecular biology, Medical research

## Abstract

Symptoms of *Staphylococcus lugdunensis* infection are often similar to those of *Staphylococcus aureus* infection, including skin and soft-tissue lesions, bacteremia and infective endocarditis. Despite the severity of these infections, *S. lugdunensis* is regarded as a less important pathogen than drug-resistant *S. aureus*. To investigate its ability to cause infectious diseases, a methicillin-resistant *S. lugdunensis* (MRSL) strain JICS135 was isolated from a patient with bacteremia and subjected to whole genome sequencing. Similar to most strains of methicillin-resistant *S. aureus* (MRSA), this MRSL strain possessed the staphylococcal cassette chromosome *mec* (SCC*mec*) located close to the origin of replication. However, the SCC*mec* in this MRSL strain, with three *ccr* complexes, was structurally unique and currently untypable. Moreover, the SCC*mec* of this MRSL strain was found to carry two genes encoding microbial surface components recognizing adhesive matrix molecules (MSCRAMM)-like proteins accompanied by glycosyl transferases, one of which may have been derived from *S. aureus* and the other from *S. epidermidis*, indicating that this MRSL evolved to carry virulence factors from other staphylococci. The emergence of this strain, the first MRSL strain whose genome has been sequenced completely, may be of public concern.

## Introduction

In 1988, two new coagulase-negative species, *Staphylococcus lugdunensis* and *S. schleiferi*, were isolated from human clinical specimens^1^. One of these human pathogens, *S. lugdunensis*, initially isolated from an axillary lymph node sample^1^, has become a coagulase-negative *Staphylococcus* species (C-NS) of significant interest^2^. Similar to *S. aureus*, *S. lugdunensis* is a skin-commensal species and a pathogen responsible for hospital- and community-acquired infections^2^. *S. lugdunensis* causes skin and soft tissue infections, bloodstream infections, and infective endocarditis^3^.

Methicillin-resistant *S. lugdunensis* (MRSL) was first isolated in 2003 from premature neonate in Singapore with a catheter-associated bloodstream infection^4^. Since then, MRSL has been isolated from patients in several countries, including Ethiopia^5^, Hong Kong^6^, Italy^7^, Singapore^8^, Taiwan^9–12^ and the USA^13,14^. A prospective study in Singapore^8^ showed that five (4.7%) of 106 clinical isolates of *S. lugdunensis* collected from 2004 to 2006 were resistant to methicillin and harbored *mecA*. The *mecA* gene encodes an alternative penicillin-binding protein 2 (PBP 2a) which has lowered affinity to β-lactam reagents, preventing bacterial growth retardation by the drugs. Dissemination of MRSL was detected in nephrology centers in Hong Kong; of 252 patients, 21 (8.3%) were MRSL carriers^6^. Subsequently, MRSL was isolated from three (42.8%) of seven patients with *S. lugdunensis* bacteremia in Japan^15^ and from seven (46.6%) of 15 patients with *S. lugdunensis* bacteremia in Iraq^16^. The increased recognition of MRSL among *S. lugdunensis* isolates suggests an emerging public health problem. A molecular epidemiological study demonstrated that MRSL isolates containing staphylococcal cassette chromosome *mec* (SCC*mec*) V structure while harboring an additional *ccrAB2* locus were emerging in central Taiwan^10^. To date, however, the complete genome sequence of a MRSL strain has not been determined. The present study reports the comparative whole genome analysis of a clinical MRSL isolate from Japan that caused a bloodstream infection. The structure of its SCC*mec* was determined and its characteristics analyzed.

## Methods

### Statement on ethics control and appropriateness of the experiments

All of the methods and the experimental protocols employed in this study were performed in accordance with relevant guidelines and regulations, and were approved by the Juntendo University School of Medicine Research Ethics Committee (permission #2019041) and the Saiseikai Yokohamashi Tobu Hospital Ethics Committee (permission #2018065). Informed consent was obtained from all participants. Prior to the start of this study, all researchers who performed these experiments had completed an ethics training course provided by the Association for the Promotion of Research Integrity, Tokyo, Japan.

### Bacterial isolates and patient characteristics

JICS135 was isolated from one of two sets of blood culture taken from an inpatient in 2014. The inpatient was a 77 year-old man with chronic kidney disease who required a long-term internal catheter. Blood cultures were processed using the BacTAlert system (bioMe´rieux, Basingstoke, UK) at Saiseikai Yokohamashi Tobu Hospital in Japan. Identification and minimal inhibitory concentrations (MICs) of antibiotics were determined by DxM 1096 MicroScan WalkAway (Beckman Coulter, U.S.) based on Clinical and Laboratory Standards Institute (CLSI) guidelines (M100S, 26^th^ edition).

### DNA manipulation and species identification

Strain JICS135 was grown on sheep blood agar (Kyokuto Pharmaceutical Industrial Co., Ltd., Japan), subjected to Microflex Biotyper matrix-assisted laser desorption ionization/time of flight mass spectrometry (MALDI-TOF MS)^17^ and identified by comparison with a database complete as of March 2018 (Bruker, Billerica, MA, USA). The complete genome determination performed in this study, followed by comparisons of its 16S ribosomal RNA gene sequence with identical sequences in the database and average nucleotide identity (ANI) analysis^18,19^ employing ANI calculator^20^ in EZbiocloud homepage (https://www.ezbiocloud.net/tools/ani) confirmed that JICS135 was *S. lugdunensis*.

### Genome sequencing, annotation and comparisons with other *S. lugdunensis* strains

**Figure 1:**
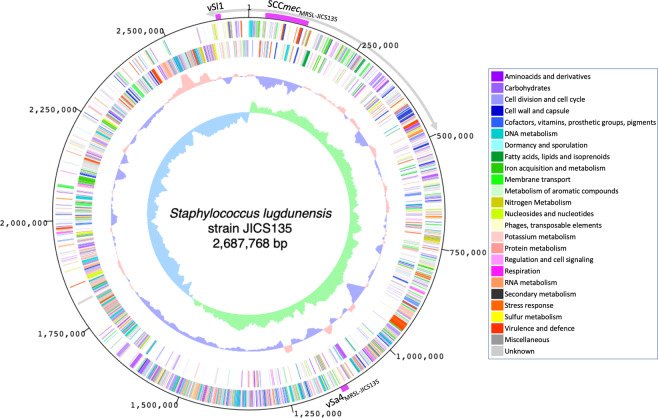
Functional genomic organization of the chromosome of *Staphylococcus lugdunensis* strain JICS135. The first and second outermost circles show open reading frames on the plus and minus strands, respectively. Colors are explained in a table to the right of the figure. The third circle shows G + C contents, with purple indicating higher than average, and the fourth circle shows GC-skew (light green means higher than average). Positions of SCC*mec*_MRSL-JICS135_ (Fig. 5) and νSa4_MRSL-JICS135_ and νSl1 (Fig. 6) are also indicated. A gray arc with arrowheads outside represents a region with low homology to *S. aureus* genomes as shown in Fig. 2.

**Figure 2:**
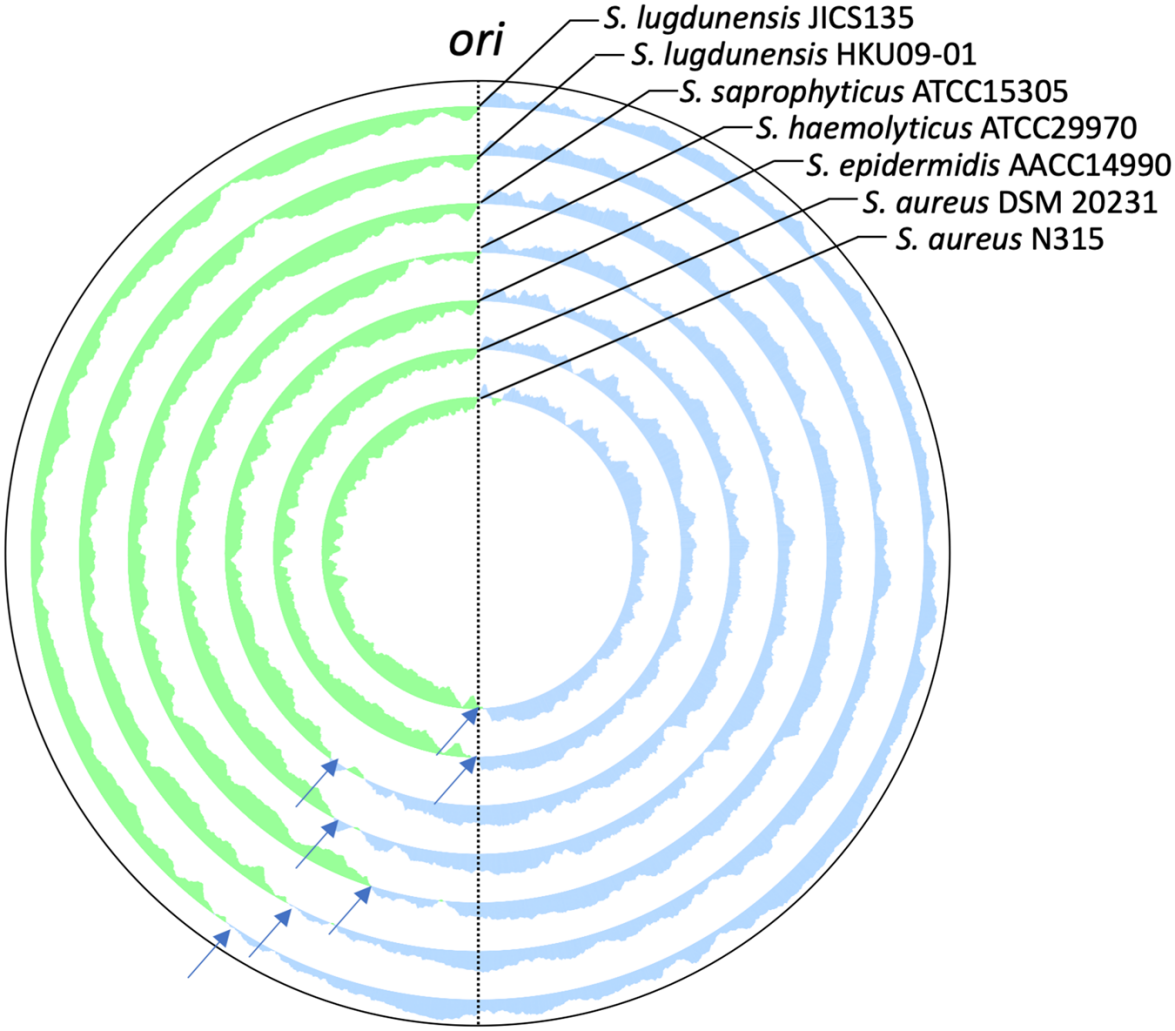
Comparison of chromosomal GC-skew of *Staphylococcus lugdunensis* strain JICS135 to other staphylococci. Arrows indicate positions where the GC-skew trend change near presumable replication termination sites. Changes in GC-skew values of *S. aureus* genomes are mostly symmetric across the vertical axis on the genome map, whereas those of coagulase-negative staphylococci including *S. lugdunensis* JICS135 are not.

**Figure 3:**
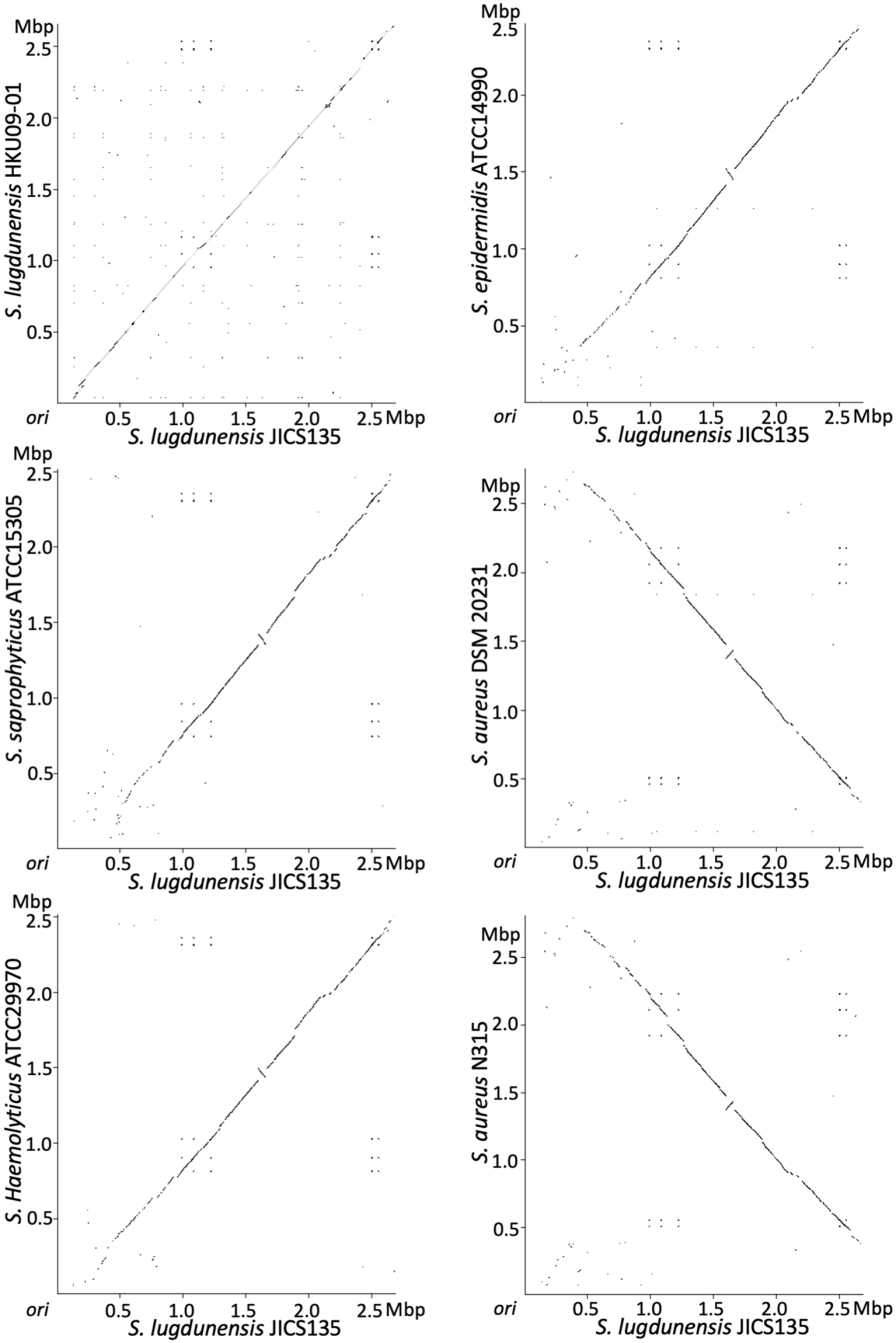
Homologous regions of JICS135 to other staphylococcal chromosomes by dot-plots. When compared to *S. aureus* genomes, large-scale rearrangement of chromosome is seen in coagulase-negative staphylococci including *S. lugdunensis* JICS135.

The genomic DNA of JICS135 was subjected to whole-genome sequencing using PacBio RS II (Pacific Biosciences, Menlo Park, CA). A total of 1163.8 Mbp (433x coverage) sequencing reads were assembled with HGAP 2.0^21^, followed by circularization with Minimus 2^22^. The RAST automated annotation servers^23^ were used for primary coding sequence (CDS) extraction and initial functional assignment. The CDS annotations were confirmed by one-to-one visual comparisons on *InSilico* Molecular Cloning (IMC) software (In Silico Biology, Inc., Kanagawa, Japan), which assists in evaluating the prevalence of the annotated sequence by comparison of each CDS with its homologues registered in databases. IMC software as also used for circular genome display and comparative analyses of the JICS135 genome with the genomes of the *S. lugdunenis* strains HKU09-01 and N920143 (Figs. 1–3). The sequence and annotation have been deposited in the databases with accession number AP021848.

### Other computer-based genome analyses

Multilocus sequence typing (MLST) was determined by depositing the complete genome sequence of JICS135 in the Center for Genomic Epidemiology (CGE) website^24^. *S. aureus* virulence genes were identified using VirulenceFinder 2.0^25^ of the CGE server with thresholds of 90% nucleotide sequence identity and 60% minimum length. Drug resistant genes were initially identified by ResFinder 3.2^26,27^ of the CGE server, followed by one-to-one visual inspection of annotated genes. Phylogenetic relationship among sequenced *S. lugdunensis* strains was analyzed by CSI Phylogeny 1.4^28^ of the CGE server, that is based on single nucleotide polymorphism (SNP) among genomes, allowing to draw a maximum-likelihood phylogenetic tree^29^. With a NEWICK-format file from result of analysis by the CSI Phylogeny, a tree was re-drawn as a radial layout with centered root by FigTree ver. 1.4.4 software (http://tree.bio.ed.ac.uk/, personally distributed by Professor Andrew Rambaut at Institute of Evolutionary Biology, University of Edinburgh). The IMC software described above was employed for GC-contents, GC-skew analyses and dot plots to identify homologous regions in two genomes.

## Results

After complete genome determination of strain JICS135, the sequence was subjected to average nucleotide identity (ANI) analysis with whole genome sequences of *S. lugdunensis* strains HKU09-01^30^ and N920143^31^. The Ortho ANIu values^20^ to the genomes were 99.30% and 99.41%, respectively. These values are sufficient to conclude that the strain JICS135 is *S. lugdunensis*.

Figure 1 shows the overall features of the entire genome of MRSL strain JICS135. The chromosome of this strain contained 2,687,768 base pairs, encoding 2,498 proteins, six sets of ribosomal RNA genes and 61 transfer RNA genes. The GC contents (33.72%) did not differ markedly from those of other genomes of well-known human-pathogenic staphylococci, including *S. lugdunensis* reference genome strain HKU09-01^30^ (33.87%), *S. saprophyticus* type strain ATCC 15305^32^ (33.24%), *S. haemolyticus* type strain ATCC29970 (32.93%) [accession #CP035291], *S. epidermidis* type strain ATCC 14990 (32.25%) [accession #CP035288], and *S. aureus* type strain DSM 20231^33^ (32.86%). Plasmids were not detected in JICS135, indicating that all drug-resistance genes are on its chromosome. Its MLST was found to be ST3.

Interestingly, direction of gene transcription of the JICS135 genome was not symmetric with respect to the vertical axis of the genome map, with the change in direction observed around 7 o’clock, a finding supported by the GC-skew values (Fig. 1). This finding suggests that the replication termination site of JICS135 is located around the 7 o’clock position on the genome map. Genomes of *S. lugdunensis* HKU09-01, *S. saprophyticus* ATCC 15305, *S. haemolyticus* ATCC 29970 and *S. epidermidis* ATCC 14990 showed similar biased GC-skew, whereas *S. aureus* strains DSM 20231 (without SCC*mec*) and N315 (with SCC*mec*)^34^ were symmetric across the vertical axis (Fig. 2). The genomes of coagulase-negative staphylococci including *S. lugdunensis* JICS135 showed large-scale chromosomal rearrangements when compared with the genomes of *S. aureus* strains, and a non-homologous region of JICS135 to genomes of other staphylococcal species was found across the replication origin (*ori*) (Fig. 3). Approximate position of the non-homologous region is indicated in Fig. 1 as an arc of outer circle with arrows.

JICS135 contained three drug-resistance genes, the β-lactam-resistant genes *mecA* and *blaZ*, and an aminoglycoside-resistant gene *aac(6*′*)-aph(2*″*)*. The nucleotide sequence of *mecA* in JICS135 was 99.90% identical to the *mecA* sequence of *S. aureus* strain N315. The minimum inhibitory concentrations (MIC) of various antibiotics to JICS135 are shown in Table 1. JICS135 was resistant to oxacillin and methicillin, suggesting its resistance to all β-lactams tested. JICS135 was susceptible to antibacterial agents showing activity against MRSA, such as linezolid (LZD), vancomycin (VCM), daptomycin (DAP), and arbekacin (ABK), as well as to levofloxacin (LVFX), but showed intermediate resistance to gentamicin. These results support the finding, that the JICS135 genome encoded only β-lactam- and aminoglycoside-resistance genes.

**Table 1:** Minimum Inhibitory Concentration (MIC) distribution and antimicrobial susceptibility test^*a*^ in JICS135.

Antimicrobial agents	MIC (μg/mL)	Result
Oxacillin	>2	R
Cefoxitin	>4	R
Benzylpenicillin	>8	R
Ampicillin	>8	R
Cefazolin	≤8	R^*b*^
Cefmetazole	≤16	R^*b*^
Ampicillin/Sulbactam	≤8	R^*b*^
Imipenem	≤1	R^*b*^
Gentamicin	>8	R
Arbekacin	≤1	^*c*^
Erythromycin	≤0.5	S
Clindamycin	≤0.5	S
Minocycline	≤2	S
Levofloxacin	≤0.5	S
Sulfamethoxazole/Trimethoprim	≤1/19	S
Fosfomycin	16	I
Rifampicin	≤0.5	S
Vancomycin	≤0.5	S
Teicoplanin	≤2	S
Linezolid	≤1	S
Daptomycin	≤0.25	S
Mupirocin	≤256	S

^*a*^Breakpoints are according CLSI guidelines (M100S, 26^th^ edition). ^*b*^All β-lactam antimicrobial agents were converted to resistant. ^*c*^Not determined.

**Figure 4:**
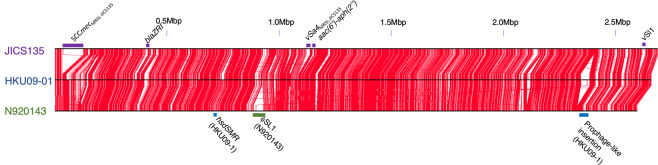
Genome rearrangement map of *Staphylococcus lugdunensis* strain JICS135 compared with *S. lugdunensis* strains HKU09-01 and N920143. Regions of >90% nucleotide identity are shown with red lines, illuminating gaps representing regions specific to each strain. Major insertions of JICS135 are indicated in the figure.

**Figure 5:**
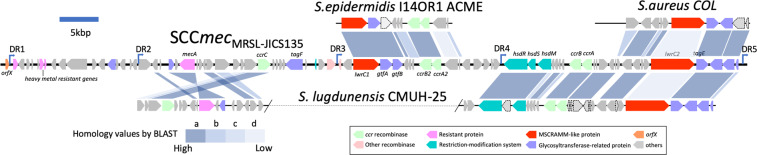
Structure of SCC*mec*_MRSL-JICS135_ compared with closely-related structures. Arrows indicates *orfs* and their directions. Three sets of *ccr* genes, responsible for the integration and excision of SCC, were identified. The *mecA* gene in SCC*mec*_MRSL-JICS135_ was the only drug resistant determinant. Two novel genes, *lwrC1* and *lwrC2*, characteristic of MSCRAMMs, were accompanied by glycosylation-related genes. The sequences of the direct repeats (DR) were: DR1, 5′-GAAGGGTATCATAAATAA-3′; DR2, 5′-GAAGCGTATCATAAATAA-3′; DR3, 5′-GAAGCATATCATAAATGA-3′; DR4, 5′-GAAGCATATCATAAGTGA-3′; and DR5, 5′-GAAGCGTATCATAAGTGA-3′. Closely-related structures found by BLAST analyses were aligned in parallel with colors representing homology: a, 0.0 ≤ e-value <1.0E-100, overlap ≥ 90.0%, identity ≥ 20.0%; b: 1.0E-100 ≤ e-value <1.0E-50, overlap ≥ 40.0%, identity ≥ 20.0%; c: 1.0E-100 ≤ e-value <1.0E-10, overlap ≥ 30.0%, identity ≥ 20.0%; d: 1.0E-10 ≤ e-value <1.0E-2, overlap ≥ 20.0%, identity ≥ 20.0%.

Figure 4 shows the chromosomal regions of JICS135 similar to those of two other *S. lugdunensis* strains, HKU09-01^30^ and N920143^31^. The gapped regions appearing in each strain are candidates of specific insertions occurring in its genome. JICS135 contained a large insertion, which was absent from strains HKU09-01 and N920143. This insertion was located close to the origin of replication of the JICS135 genome. This insertion included complexes of genes encoding the methicillin-resistant determinant *mec* and the DNA recombinase *ccr*, clearly indicating that this domain is the Staphylococcal Cassette Chromosome *mec* (SCC*mec*), which confers β-lactam resistance onto staphylococcal species^35^. Figure 5 illustrates the structure of the SCC*mec* of strain JICS135, which has been designated SCC*mec*_MRSL-JICS135_. This region had direct repeats at both ends, located exactly at the boundaries of the inserted region of the JICS135 genome (Fig. 4). The distance between the repeats at the ends was 92,958 bps. Other repeats were observed in the middle parts of the SCC*mec*_MRSL-JICS135_ and *ccr* complexes, suggesting that SCC*mec*_MRSL-JICS135_ was formed by multiple insertions of SCCs of different origins. Due to its complicated structure, we were unable to type SCC*mec*_MRSL-JICS135_ using established procedures^36^. SCC*mec*_MRSL-JICS135_ contained a *mecA* gene, which was not flanked by the sensor gene *mecR* or the repressor gene *mecI*, but no other determinants of drug resistance. Rather, SCC*mec*_MRSL-JICS135_ contained two genes encoding large proteins similar to staphylococcal microbial surface components recognizing adhesive matrix molecules (MSCRAMMs)^37–39^. One of these, designated *lwrC1* (gene 1 of *S*. *lugdunensis* cell wall-anchored with specific repeats in cassette chromosome), encoded a protein containing repeats of the sequence STSDSESHSDSESDSDSE, whereas the other, designated *lwrC2*, encoded a protein containing repeats of the sequence SDADSD (where S, T, D, E, A and H represent serine, threonine, glutamine, glutamic acid, alanine and histidine, respectively). The C-termini of both of these products possessed LPXTG cell wall sorting signals^40,41^, suggesting that they attach to molecules composed of cell surfaces of infected human tissues. Interestingly, the *lwrC1* and *lwrC2* genes were accompanied by transglycosylases and related genes. The *lwrC1* gene was flanked by homologues of *gtfA* and *gtfB*, which are required for the glycosylation of a *gspB* gene product and enhances attachment of *Streptococcus gordonii* to platelets^42^. Similar glycosylation of a *sraP* gene product of *S. aureus* enhances its attachment to host tissues^43^. The *lwrC2* gene is also located close to transglycosylation-related genes, suggesting that the *lwrC2* products may interact with tissues.

MRSL strains isolated in Hong Kong and Taiwan were found to have SCC*mec*s with structures similar to SCC*mec*_MRSL-JICS135_^44,45^. Draft genome information has indicated that SCC*mec*_6756_ and SCC*mec*_5580_^44^ each possesses three *ccr* complexes, two MSCRAMM-like genes and glycosyltransferases, although there were significant differences in regions between *orfX* and the *mec* complex. SCC*mec*_6756_ and SCC*mec*_5580_ have been designated SCC*mec* types V.4.1.3 and V.4.2.2, respectively. SCC*mec*_MRSL-JICS135_ was less similar to SCC*mec*s of *S. lugdunensis* strains CMUH-22 and CMUH-25^45^ than to SCC*mec*_6756_ and SCC*mec*_5580_, but was highly homologous to a region between the *hsdR* gene encoding a restriction endonuclease of a restriction-modification system and the downstream end of SCC*mec*_MRSL-JICS135_. Because the sequences of SCC*mec*_6756_ and SCC*mec*_5580_ have not been available in databases, a comparison of structure of the SCC*mec* of strain CMUH-25 with that of SCC*mec*_MRSL-JICS135_ is shown in Fig. 5. Other analyses have shown that the region including *lwrC1* was most similar to part of the arginine catabolic mobile element (ACME) region of *S. epidermidis* strain I14OR1^46^, whereas the *lwrC2* region was most similar to the SCC*mec*s of *S. lugdunensis*, followed by part of the SCC*mec* of *S. aureus* strain COL^47^. Indeed, the product of the *lwrC1* gene was most similar to the SdrH protein of *S. epidermidis* whereas the product of the *lwrC2* gene was highly similar to a hypothetical protein of *S. aureus*. These findings suggest that SCC*mec*_MRSL-JICS135_ consists of multiple domains originating from other staphylococcal species.

**Figure 6:**
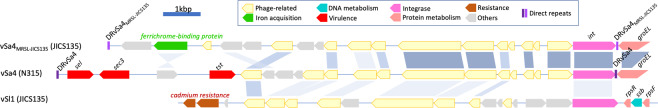
Structure of νSa4_MRSL-JICS135_ and νSa1 in comparison with νSa4 of *S. aureus* strain N315. The sequence of direct repeats for *S. aureus* νSa4 (DRνSa4) was 5′-GTTTTACATCATTCCCGGCAT-3′, whereas that for *S. lugdunensis* DRνSa4_MRSL-JICS135_ was 5′-TTTTACATCATACCTGGCAT-3′. The parallelogram with colors representing homology values are the same as those in Fig. 4.

In addition to SCC*mec*_MRSL-JICS135_, JICS135 had at least four other inserted regions relative to strains HKU09-01 and N920143, containing *blaZRI*, νSa4_MRSL-JICS135_*, aac(6*′*)-aph(2″)* and νSl1 (Fig. 4). The *blaZRI* gene confers resistance to β-lactams, and the insertion contained a transposase similar to Tn554, indicating that the drug-resistance gene was inserted together with the transposon, as the *blaZRI-*Tn554 combination is widely seen in staphylococci. The aminoglycoside-resistance gene *aac(6*′*)-aph(2*″*)* was accompanied by IS256 (Fig. 4), which is also widely seen in staphylococci. JICS135 also contained two genomic islands, νSa4_MRSL-JICS135_ and νSl1 (Figs. 4 and 6), similar to the *S. aureus* genomic island νSa4^48^, which often carries the *tst* gene encoding the protein toxic shock syndrome toxin 1 (TSST-1). In comparison with νSa4 of *S. aureus* strain N315, which contains the genes *sel*, *sec. 3* and *tst*, encoding the superantigens TSST-1 and staphylococcal enterotoxins L and C3, respectively^34^, νSa4_MRSL-JICS135_ contained a gene encoding a ferrichrome-binding protein, which is involved in iron acquisition, and νSl1 contained cadmium resistance genes (Fig. 6). The sequence of an integrase for νSa4_MRSL-JICS135_ was 96% identical to that of νSa4 of *S. aureus* strain N315, with sequences for the direct repeats at both ends included in those of N315 (Fig. 6), strongly suggesting that νSa4_MRSL-JICS135_ and *S. aureus* νSa4 shared a common origin. In contrast to νSa4_MRSL-JICS135_, the integrase for νSl1 had only 29% sequence identity to that of νSa4 of *S. aureus* strain N315. We failed to identify direct repeats at both ends. Although νSl1 and νSa4 of *S. aureus* strain N315 had several genes in common, their lineages may differ. Because a database search showed that *S. lugdunensis* strains Klug93G-4^49^, FDAARGOS141, FDAARGOS377, and FDAARGOS381, with accession numbers CP014022, CP023539 and CP023970, respectively, had elements 99.9% identical to that of JICS135, we designated this element as νSl1 (i.e. the first ν element identified in *S. lugdunensis*). *S. lugdunensis* strains HKU09-01 and N920143 did not possess integrases identical to those for νSa4_MRSL-JICS135_ and νSl1.

A comparative analysis also revealed unique insertions in strains HKU09-01 and N920143. The former possessed a restriction-modification system (*hsdSMR*) and a prophage-like insertion with integrase and phage component genes, whereas the latter possessed an insertion of prophage ϕSL1 (Fig. 4). The JICS135 genome did not have *hsdSMR* at the site corresponding to that of HKU09-01; however, this gene cluster was found in SCC*mec*_MRSL-JICS135_ (Fig. 5).

**Figure 7:**
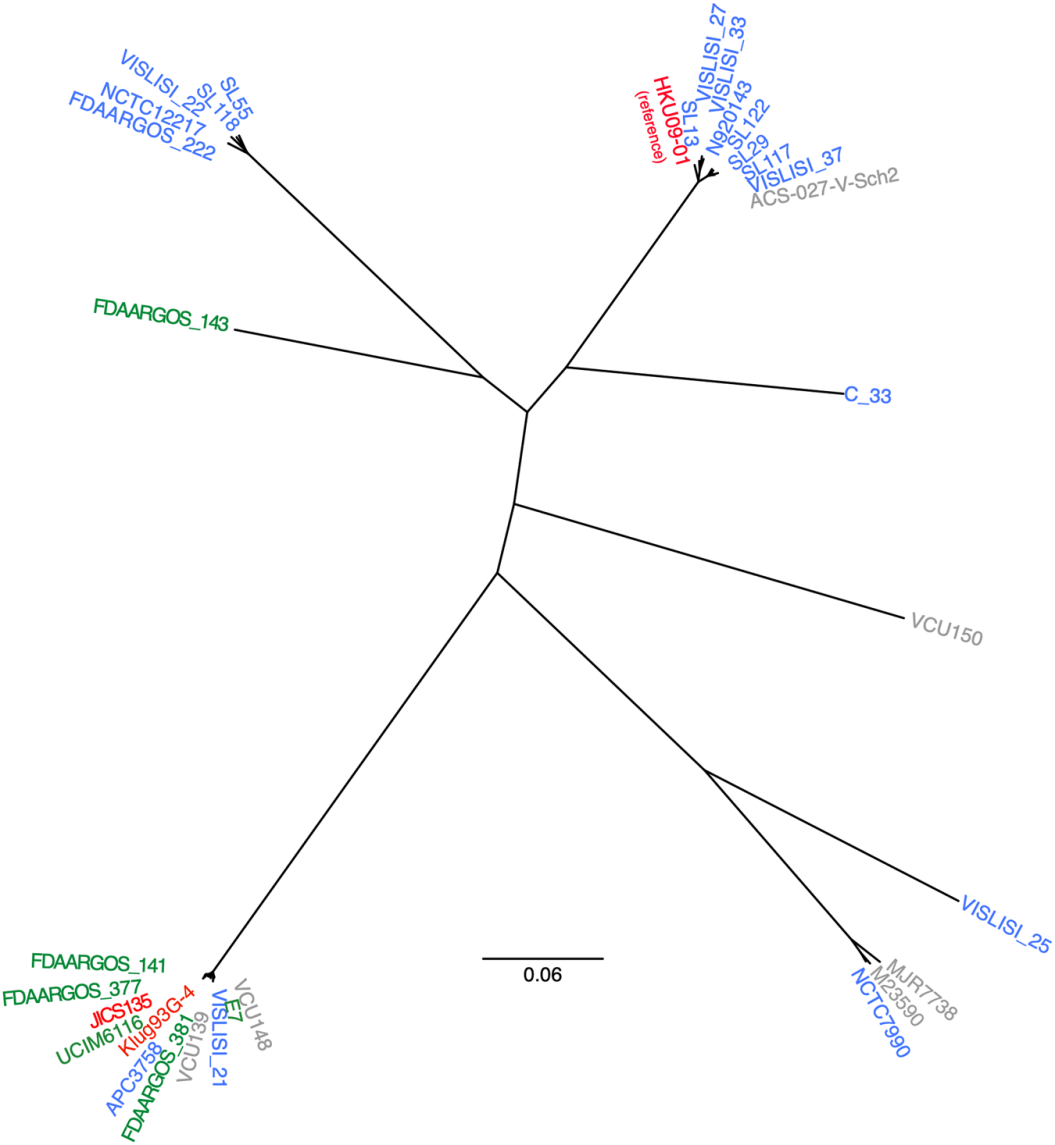
Whole genome-wide phylogenetic relationship among *S. lugdunensis* strains. Maximum-likelihood tree based on SNPs extracted from the genomes by mapping them to reference sequence of *S. lugdunensis* strain HKU09-01^30^ is presented. The branch length indicates proportions of SNPs to the total 23,055 ones called among the 33 genomes (refer to the scale bar). *S. lugdunensis* strains that whole genome sequences are available in databases, or those of strains HKU09-01, M23590 (BioSample #SAMN00139437), N920143^31^, VCU139 (BioSample #SAMN02436593), VCU148 (BioSample #SAMN00116837), VCU150 (BioSample #SAMN00116873), ASC-027-V-Sch2 (BioSample #SAMN02463890), UCIM6116 (BioSample #SAMN02381740), MJR7738 (BioSample #SAMN03948580), FDAARGOS_141^57^, FDAARGOS_143^57^, FDAARGOS_222^57^, FDAARGOS_377^57^, FDAARGOS_381^57^, VISLISI_21^58^, VISLISI_22^58^, VISLISI_25^58^, VISLISI_27^58^, VISLISI_33^58^, VISLISI_37^58^, C_33^58^, Klug93G-4^49^, NCTC7990 (accession #LS483312), NCTC12217 (BioSample #SAMN06177169), E7 (BioSample #SAMN10735326), SL13 (accession #CP041722.1), SL29 (accession #CP041723.1), SL55 (accession #CP041724.1), SL117 (accession #CP041725.1), SL118 (accession #CP041726.1), SL122 (accession #CP041727.1), APC3758 (accession #CP038807.1) and JICS135 (this study) were subjected to CSI Phylogeny 1.4^28^ with parameters of default values (minimum depth at SNP positions: 10, relative depth at SNP positions: 10, minimum distance between SNPs: 10, minimum SNP quality: 30, minimum read mapping quality: 25, minimum Z-score: 1.96 with ignoring heterozygous SNPs). Strains isolated in Asia, Europe and north America are indicated in red, blue and green, respectively. Strains without information about locations of isolation are indicated in gray.

In order to see relationship among sequenced *S. lugdunensis* strains, a whole genome-wide phylogenetic analysis based on single nucleotide polymorphism (SNP) was performed (Fig. 7). JICS135 was relatively close to strain Klug93G-4 isolated in Hong Kong. The two Asian isolates JICS135 and Klug92G-4 (red) were also phylogenetically close to the north American ones (green), whereas the European isolates (blue) seemed to form a few clades that had distance from the one with JICS135 and some north American strains. On the other hand, *S. lugdunensis* whole genome sequence reference strain HKU09-01 isolated in Hong Kong belonged to one of the European clades. In addition to JICS135, only UCIM6116 had *mecA* and *ccrC* genes, suggesting that the strain has SCC*mec* among the strains shown in Fig. 7. However, the sequence of UCIM6116 lacked *ccrAB, ccrA2B2, lwrC1* and *lwrC2*, indicating that the strain does not have similar element to SCC*mec*_MRSL-JICS135_.

## Discussion

The complete sequencing of the genome of methicillin-resistant *S. lugdunensis* strain JICS135 showed that the size of its genome was approximately the same as other coagulase-negative staphylococci, and smaller than that of *S. aureus*. Due mainly to the insertion of SCC*mec*, the genome of JICS135 was larger than that of the genomes of methicillin-susceptible *S. lugdunensis* strains described to date. Unlike *S. aureus* genomes, the transcriptional direction of genes was not symmetric across the vertical axis of the genomic map of JICS135, suggesting that the replication termination site is not located opposite the site of the origin of replication on the circular chromosome, but at about the 7 o’clock position on the genome map, a finding supported by GC-skew results. This asymmetry was initially thought to be due to the insertion of SCC*mec*_MRSL-JICS135_ slightly downstream of the origin of replication. Similar findings, however, were observed in other *S. lugdunensis* genomes without SCC*mec*s, suggesting that insertion of SCC*mec*_MRSL-JICS135_ was not responsible for this asymmetry. *S. haemolyticus* strain JCSC1435 also shows asymmetry in GC-skew^50^, with similar phenomena observed in other coagulase-negative staphylococci (Fig. 2). Dot plots have shown that coagulase-negative staphylococci had large-scale genome inversions when compared with *S. aureus* genomes. It was also notable that regions around the origins of replication of *S. aureus* genomes and JICS135 were not homologous, with these regions extending 100 kbp upstream and 500 kbp downstream of the origin of the JICS135 genome, as shown in Fig. 1. Because the non-homologous region was shifted to the right side of the genome map, that side could include sequences that lead to the GC-skew bias. We also found that frequently-transcribed genes that can affect replication speed, such as those encoding ribosomal RNAs, ribosomal proteins and tRNAs, located on the right side of the genome map were concentrated in JICS135. In contrast, those located on the left side were scattered, delaying replication of the left side relative to the right side and leading to the replication termination site being located at about the 7 o’clock position.

ST3 was found to be the most frequently isolated (20.7%) *S. lugdunensis* MLST in France, Belgium and Slovenia, but MRSL was not found^51^. In contrast, Taiwan, ST6 (19.0%)^52^ and ST38 (77.8%)^53^ were the most common MRSLs in Taiwan. Our finding, that JICS135 belonged to ST3, indicates that the JICS135 lineage is distinct from the strains isolated in other countries.

In contrast to other *S. lugdunensis* genomes, the JICS135 genome apparently contained no prophages, as no insertions 40–50 kbp in length with phage integrase accompanied by major phage component genes were detected in JICS135. However, JICS135 had several insertions of mobile genetic elements, the most striking being as large as 93 kbp of the SCC*mec* element, SCC*mec*_MRSL-JICS135_, close to the origin of replication. The insertion of SCC*mec*_MRSL-JICS135_ occurred in *orfX* gene, encoding 23S rRNA (pseudouridine [1915]-N[3])-methyltransferase RlmH^54^, as seen in other staphylococcal species. SCC*mec*_MRSL-JICS135_ also contained a *mec* complex and *ccr* recombinase genes. Despite their common features, the structure of SCC*mec*_MRSL-JICS135_ differs significantly from those found in other Staphylococci. SCC*mec*_MRSL-JICS135_ was as large as 93 kbp in size and contained multiple sets of *ccr* recombinases and other unique features. We found that SCC*mec*_MRSL-JICS135_ contained the *lwrC1* and *lwrC2* genes, encoding large cell-wall anchored proteins with unique repeats, a feature observed in MSCRAMMs, surface adhesive proteins typically found in *S. aureus*, suggesting that the products of the *lwrC1* and *lwrC2* genes likely play important roles in attachment to host cells and tissues^37^. This hypothesis is consistent with the finding that JICS135 was isolated from a patient with bloodsteam infection. Moreover, the *lwrC1* and *lwrC2* genes were accompanied by transglycosylases and related genes. In other genera, these glycosylated proteins are secreted by specific mechanisms involving SecA2 and SecY2 that are distinct from general secretory mechanisms. These secreted proteins subsequently attach covalently to the bacterial cell walls, increasing their affinity to platelets^42^. Because strain JICS135 does not contain genes encoding SecA2 or SecY2, the mechanism by which the *lwrC1* and *lwrC2* gene products translocate through the cytoplasmic membrane is unclear. Although the targets of these gene products have not been determined, SCC*mec*_MRSL-JICS135_ containing these MSCRAMM genes may increase the virulence of *S. lugdunensis* strains. Investigations to identify the molecules targeted by the *lwrC1* and *lwrC2* gene products are ongoing.

Analysis also revealed that the *lwrC1* locus has the highest homology to *S. epidermidis*, whereas the *lwrC2* locus is most similar to *S. aureus*, suggesting that SCC*mec*_MRSL-JICS135_ is a hybrid of staphylococcal strains resulting from multiple gene crossovers. Because both *S. epidermidis* and *S. aureus* are included in normal human flora, MSSL strains can acquire both higher virulence and drug resistance by the incorporation of elements such as SCC*mec*_MRSL-JICS135_. In addition to the *lwrC1* and *lwrC2* genes, other MSCRAMM genes have been detected in *S. lugdunensis*^31^. The combination of these MSCRAMMs and SCC*mec* can enhance the virulence of MRSL strains. Further analyses will be required to prove whether SCC*mec*_MRSL-JICS135_ confers higher affinity to fibronectin to JICS135 and the enhanced affinity leads to increase of virulence of the strain.

Three genes responsible for antibiotic resistance were identified in JICS135. The *mecA* gene in SCC*mec*_MRSL-JICS135_ was not accompanied by *mecR* and *mecI*, whereas the β-lactamase *blaZ* gene was accompanied by the sensor gene *blaR* and the repressor gene *blaI*. These findings suggested that *blaZ* gene expression correlates with the concentration of β-lactam reagents, and that *mecA* gene expression is under the control of β-lactams via *blaR*^55^. The MICs for β-lactams indicate that of JICS135 is resistant to these antibiotics, which may be a consequence of the combined effects of *mecA* and *blaZ*. The *aac(6*′*)-aph(2*″*)* gene, which is responsible for aminoglycoside resistance, was also functional, because the MIC for gentamycin indicates intermediate resistance of JICS135. Similar to many MRSA strains, the *aac(6*′*)-aph(2*″*)* gene is inserted into the JICS135 genome along with the transposon Tn*554*, indicating inter-species horizontal transfer among staphylococci, probably under selective pressure of aminoglycoside reagents.

No other known *S. aureus* virulence factors other than these MSCRAMM proteins were detected in *S. lugdunensis* JICS135 genome. Genetic methods are required to identify *S. lugdunensis* genes associated with virulence. For example, virulence can be evaluated in a library subjected to transposon-insertion mutagenesis using a model organism^56^. This approach may provide more information needed to understand the pathogenicity of *S. lugdunensis*.

Because fewer people have been infected by *S. lugdunensis* than by *S. aureus* and other major coagulase-negative staphylococci, little is known about the molecular epidemiology of *S. lugdunensis* infection. This drug-resistant *S. lugdunensis* strain containing a complex SCC*mec*_MRSL-JICS135_ structure may become more widespread, suggesting the need for continuous surveys of *S. lugdunensis* isolates, including their drug resistance properties and their association with patient symptoms. Our analysis using whole genome sequences of *S. lugdunensis* in Fig. 7 showed that phylogenetically close strains to JICS135 are present. In addition to JICS135, however, only strain UCIM6116 (the sequencing has not been completed) seemed to have SCC*mec* which structure was not likely to be similar to SCC*mec*_MRSL-JICS135_. Further analyses will elucidate relationship among carriage of SCC*mec*s, their structures and sequence types of other parts of chromosomes, and the information would provide us evolutional pathways of MRSL strains.

This comparative analysis of *S. lugdunensis* genomes, including JICS135, revealed variations in their mobile genetic elements, which are responsible for the drug resistance and virulence of these strains. These findings suggested that novel types of *S. lugdunensis* strains that differ in drug resistance and virulence emerge as causes of hospital- and community-acquired infections.
